# Ipragliflozin and sitagliptin differentially affect lipid and apolipoprotein profiles in type 2 diabetes: the SUCRE study

**DOI:** 10.1186/s12933-024-02149-7

**Published:** 2024-02-08

**Authors:** Mototsugu Nagao, Jun Sasaki, Kyoko Tanimura-Inagaki, Ichiro Sakuma, Hitoshi Sugihara, Shinichi Oikawa, on behalf of the SUCRE Study Investigators

**Affiliations:** 1 Department of Endocrinology, Metabolism and Nephrology, Graduate School of Medicine, Nippon Medical School, Sendagi 1-1-5, Bunkyo-ku, Tokyo, 113-8603 Japan; 2 Graduate School of Pharmaceutical Medicine, International University of Health and Welfare, Nagahama 1-3-1, Chuo-ku, Fukuoka, 810-0072 Japan; 3 Caress Sapporo Hokko Memorial Clinic, Kita 27 Higashi 8 1-15, Higashi-ku, Sapporo, 065-0027 Japan; 4 Diabetes and Lifestyle-Related Disease Center, Fukujuji Hospital, Anti-Tuberculosis Association (JATA), Matsuyama 3-1-24, Kiyose, Tokyo, 204-8522 Japan

## Abstract

**Background:**

SGLT2 inhibitors and DPP4 inhibitors have been suggested to affect lipid metabolism. However, there are few randomized controlled trials comparing the effects on the lipid metabolism between the two types of antidiabetic drugs. The SUCRE study (UMIN ID: 000018084) was designed to compare the effects of ipragliflozin and sitagliptin on serum lipid and apolipoprotein profiles and other clinical parameters.

**Methods:**

This is a multicenter, open-label, randomized, controlled trial. Patients with type 2 diabetes (20–74 years old) with HbA1c levels of 7.0-10.5% and serum triglyceride levels of 120–399 mg/dL (1.35–4.50 mmol/L) on diet and/or oral hypoglycemic agents were enrolled. Subjects were randomized to treatment with ipragliflozin (50 mg/day, *n* = 77) or sitagliptin (50 mg/day, *n* = 83). Laboratory measurements were performed at 0, 1, 3, and 6 months of treatment.

**Results:**

Ipragliflozin and sitagliptin reduced fasting plasma glucose, glycoalbumin, and HbA1c almost equally. Ipragliflozin increased HDL-C and decreased apo E. Sitagliptin decreased TG, apo B48, CII, and CIII, but increased LDL-C. The between-treatment differences were significant for HDL-C (*P* = 0.02) and apo B48 (*P* = 0.006), and nearly significant for apo A1 (*P* = 0.06). In addition, ipragliflozin reduced body weight, blood pressure, serum liver enzymes, uric acid, and leptin, and increased serum ketones compared with sitagliptin.

**Conclusions:**

While ipragliflozin and sitagliptin showed similar effects on glycemic parameters, the effects on serum lipid and apolipoprotein profiles were different. Ipragliflozin may have an anti-atherogenic effect through modulation of HDL-C and apo E compared to sitagliptin through TG and apo B48, CII, and CIII in patients with type 2 diabetes.

**Supplementary Information:**

The online version contains supplementary material available at 10.1186/s12933-024-02149-7.

## Background

Diabetes is a major risk factor for the development of atherosclerosis and is associated with a 2- to 4-fold increased risk of cardiovascular disease [1]. Hyperglycemia, especially postprandial hyperglycemia, has been shown to cause atherosclerosis [2]. Adequate glycemic control is therefore important for the prevention of cardiovascular disease. Both sodium-glucose cotransporter 2 (SGLT2) and dipeptidyl peptidase-4 (DPP4) inhibitors are promising drugs for this purpose [3]. SGLT2 inhibitors promote urinary glucose excretion by inhibiting renal tubular SGLT2 and improve hyperglycemia in an insulin-independent manner [4]. On the other hand, DPP4 inhibitors maintain blood incretin levels by inhibiting the incretin-degrading activity of DPP4, promote insulin secretory secretion in response to blood glucose levels, and improve glucose metabolism [5]. SGLT2 inhibitors are expected to improve hyperglycemia even in the presence of insulin deficiency, while DPP4 inhibitors are effective in those with some preservation of insulin secretion [3].

Both SGLT2 and DPP4 inhibitors exhibit diverse advantages beyond their glucose-lowering properties. DPP4 inhibitors have a neutral influence on body weight, minimal risk of hypoglycemia [6], and safety in terms of major cardiovascular outcomes [7], excluding heart failure with saxagliptin [8] and possibly alogliptin [9]. Conversely, SGLT2 inhibitors provide significant benefits, including body weight reduction [10], decreased risk of atherosclerotic cardiovascular disease and heart failure [11, 12], beneficial effects on kidney function [13], and a reduction in all-cause mortality [14]. Furthermore, both agents have been shown to affect lipid metabolism. For example, SGLT2 inhibitors have been reported to increase low-density lipoprotein cholesterol (LDL-C) and high-density lipoprotein cholesterol (HDL-C) [15, 16] and DPP4 inhibitors to decrease triglycerides (TG) and remnants [17, 18]. Since dysregulation of lipid metabolism plays a key role in the development of atherosclerosis associated with diabetes [19], it is of great importance to elucidate the pleiotropic effects of these drugs on lipid and apolipoprotein profiles. This understanding is crucial in formulating an optimal treatment strategy for diabetes, particularly in conjunction with atherosclerosis. However, there are few randomized controlled trials comparing the effects of SGLT2 inhibitors and DPP4 inhibitors on lipid and apolipoprotein profiles. Here, we conducted a multicenter, randomized trial to compare the effects of a SGLT2 inhibitor (ipragliflozin) and a DPP4 inhibitor (sitagliptin) in patients with type 2 diabetes. The SUCRE study was designed to analyze the effects of ipragliflozin and sitagliptin primarily on lipid metabolism, and secondarily on glucose metabolism and other clinical parameters related to atherosclerosis, to clarify the anti-atherogenic benefits of the two drugs. The primary outcome measures were serum lipids and apolipoproteins. Secondary outcome measures included glucose metabolism parameters, body weight, systolic and diastolic blood pressure, and renal function parameters.

## Methods

### Study design

The present study was designed as a multicenter, open-label, randomized controlled trial. This study was registered in the University Hospital Medical Information Network (UMIN) as the Sodium-GlUcose Co-transporteR 2 inhibitor, Ipragliflozin, Suglat®, Effect on Lipid and Glucose Metabolism (SUCRE) Study (UMIN ID: 000018084). Recruitment and follow-up of participants took place from June 2015 to September 2019 at 14 hospitals and clinics across Japan. The study adhered to the principles of the Declaration of Helsinki and was approved by the ethics committees at Fukujuji Hospital, Japan Anti-Tuberculosis Association and, where appropriate, at each of the participating hospitals and clinics. Written informed consent was obtained from all patients.

Eligible patients were outpatients with type 2 diabetes aged 20–74 years with inadequate glycemic control (HbA1c of 7.0-10.5%) and serum TG of 120–399 mg/dL (1.35–4.50 mmol/L). Patients treated with lifestyle modification and/or antidiabetic agents, excluding insulins, GLP-1 receptor agonists, SGLT2 inhibitors, and DPP4 inhibitors, were included in the study. Glycemic control requires HbA1c variability to be stable within 1% in the absolute value over the past 2 months. Patients were ineligible if they received continuous treatment with lipid-lowering agents (statins, ezetimibe, fibrates, nicotinic acid, probucol, anion exchange resins, or eicosatetraenoic acid); if they had type 1 diabetes, proliferative retinopathy, dysuria, urinary and genital infections, chronic renal impairment (serum creatinine (Cr) > 1.5 mg/dL), severe liver dysfunction (Child-Pugh class C), severe infection, trauma, or possible pregnancy; if they have a medical history of ketosis, diabetic coma, cardiovascular disease (cerebral infarction, myocardial infarction, unstable angina, angioplasty, or heart disease, NYHA class III-IV) in the past 6 months; treated with diuretics, steroids or immunosuppressive therapy for the past 6 months; or if they were allergic to SGLT2 or DPP4 inhibitors. Patients were also ineligible if the study physicians deemed it inappropriate for them to participate.

Eligible patients were enrolled and randomized (1:1) to the ipragliflozin group or the sitagliptin group. Randomization was performed using a computer-generated randomization list generated by an independent statistician using the permuted block method with a block size of 10 and equal allocation to the two treatments. Both patients and study physicians were informed of their treatment assignment. Patients received 50 mg of ipragliflozin or 50 mg of sitagliptin once daily for at least 6 months in addition to their previous treatment. The doses were determined according to the usual doses of both drugs in Japan. The glycemic target was HbA1c < 7.0%. Study physicians were asked not to change the antidiabetic regimen during the first 3 months. Thereafter, dose escalation of sitagliptin or ipragliflozin up to 100 mg/day was allowed to achieve the glycemic target. The addition, dose titration, or discontinuation of oral antidiabetic medications other than SGLT2 inhibitors, DPP4 inhibitors, and GLP-1 receptor agonists was also allowed as needed. No medications for dyslipidemia were prescribed during the study period.

### Measurements

Venous blood and urine samples were collected under fasting conditions at 0, 1, 3, and 6 months of treatment, and the samples were transported to the central laboratory (SRL Inc., Tokyo, Japan), where venous blood was centrifuged to separate serum or plasma for biochemical measurements. Blood pressure and body weight were measured at the same time of blood sampling. Peripheral blood cell counts were measured at 0, 1, 3, and 6 months.

The primary outcome measures were serum lipids and apolipoproteins. Lipid-related parameters included serum total cholesterol (TC), HDL-C, LDL-C, and TG and apolipoprotein (apo) AI, AII, B, B48, CII, CIII, and E. Secondary outcome measures included glucose metabolism parameters [FPG, insulin, HbA1c, and glycoalbumin (GA)], body weight, systolic and diastolic blood pressure, renal function parameters such as estimated glomerular filtration rate (eGFR) and urine albumin-to-Cr ratio (UACR). Serum Cr and cystatin C (CysC) concentrations were used to calculate Cr-based eGFR and cystatin CysC-based eGFR, respectively [20, 21]. Other laboratory measurements included blood cell counts, serum liver enzymes, uric acid, electrolytes, compliments, leptin, non-cholesterol sterols, selected fatty acids, and ketones.

All the laboratory measurements were performed at a central laboratory (SRL, Tokyo, Japan) using the standard methods. Details of the methods and resources are provided in Table S1. Briefly, plasma glucose was measured by the hexokinase UV method; serum insulin concentration and HbA1c by chemiluminescence enzyme immunoassays for each; and GA by the visible absorption spectrometry enzymatic method. Serum TC and TG were measured by enzymatic methods for each; HDL-C by direct assay. LDL-C was calculated using the Friedewald formula. Apo AI, AII, B, CII, CIII, and E were measured by turbidimetric immunoassays by using corresponding individual kits. Apo B48 was measured by chemiluminescent enzyme-linked immunosorbent assay using apo B48 CLEA (Fujirebio Inc., Tokyo, Japan).

### Statistical analysis

We did not statistically determine a required sample size because the present study was exploratory with respect to serum apolipoproteins. There was no information on potential differences in changes in apolipoproteins between ipragliflozin and sitagliptin treatment at the time of protocol development. The target number of patients to be enrolled was set at 200 (100 for each group) based on a previous study that reported statistically significant changes in some of the serum apolipoproteins with sitagliptin treatment [22].

The full analysis set for the assessment of outcome variables included randomized patients who had a baseline measurement and at least one follow-up measurement and who adhere to the study protocol. We did not use measurements of FPG, insulin, and TG if the blood sample was reported to have been collected in postprandial condition or if plasma insulin was ≥ 25 µU/mL (175 pmol/L). LDL-C was not calculated if TG was ≥ 400 mg/dL. Therefore, the number of patients varied at different time points during the follow-up varied (Table S2).

The distribution of each continuous variable was tested for normality using the skewness-kurtosis test. Baseline characteristics of patients were presented as mean with standard deviation (SD) for normally distributed variables, median with interquartile range (IQR, 25th and 75th percentiles) for continuous variables with a non-normal distribution, and proportions for categorical variables. Comparisons between groups at baseline were made using the unpaired *t*-test for variables with a normal distribution, the Wilcoxon rank-sum test for continuous variables with a non-normal distribution, and Fisher’s exact test for categorical variables. A mixed-model repeated-measures analysis was used to assess the between-treatment difference in the change during treatment from baseline in an outcome parameter (dependent variable). Adjustment was made for sex, age, and baseline measurement of a parameter of interest. Fixed effects were treatment, month of visit, variables used for adjustment, and treatment by month interaction, and the random effect was patient. Month of visit was treated as a categorical variable, and an unstructured variance-covariance matrix was used. The robust method was used to estimate the standard errors to allow for potential departure from the model assumption. Changes from baseline were used as such in the mixed model analysis, except for the urine albumin-to-creatinine ratio, which had an extremely skewed distribution and was transformed to natural logarithm. Marginal means and 95% confidence intervals were estimated. The analysis of adverse events was performed on all the patients who started treatment, and the comparison was based on the actual treatment received. The occurrence of adverse events was compared using Fisher’s test. *P* values < 0.05 (two-tailed) were considered statistically significant. Statistical analysis was performed using Stata version 13 software (StataCorp LLC, College Station, TX, USA).

## Results

**Fig. 1:**
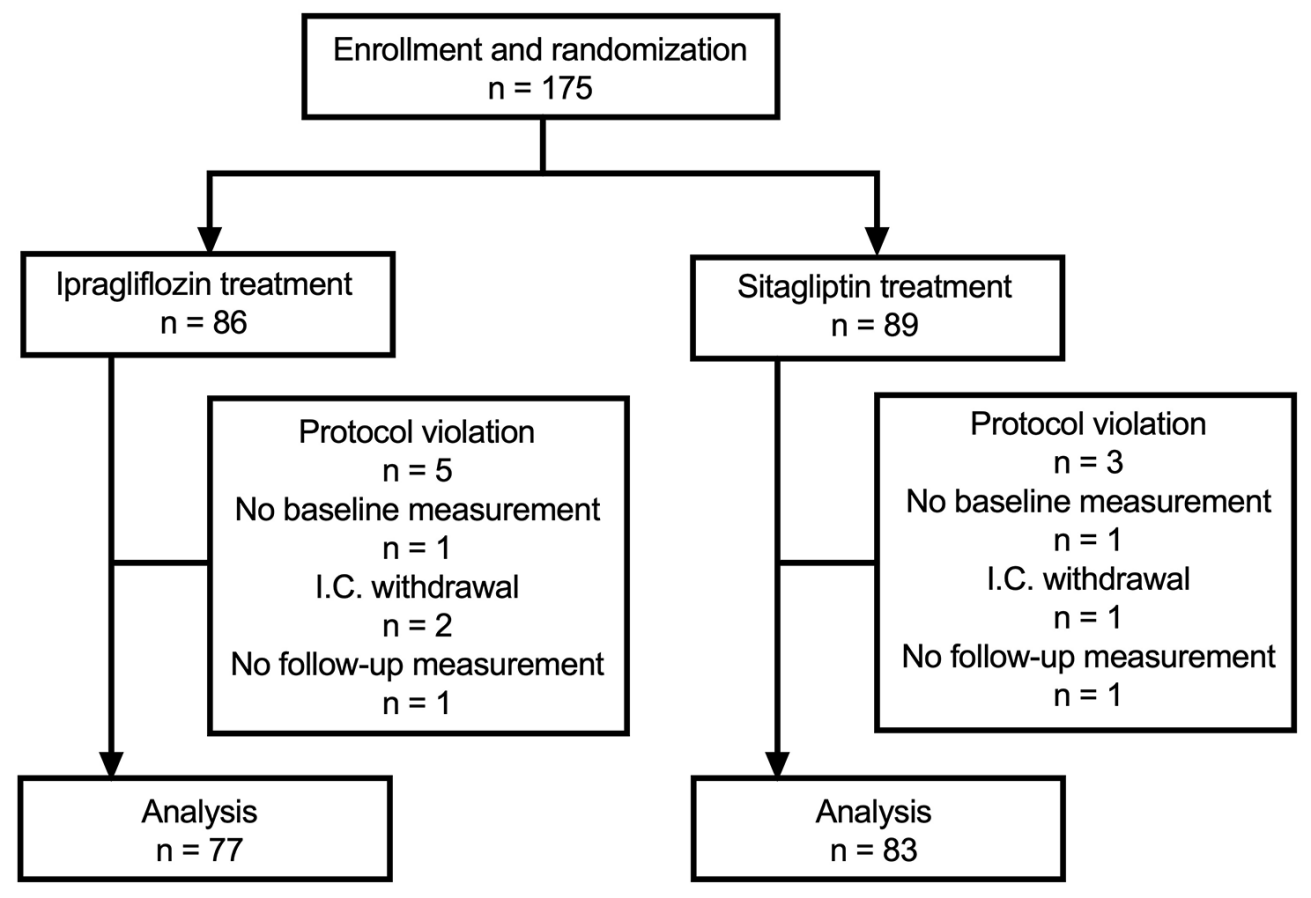
Flowchart of subject enrollment. A total of 175 patients were enrolled and randomized to ipragliflozin or sitagliptin. Of the 175 participants, 15 were excluded from the efficacy analysis due to protocol violations, no baseline measurements, withdrawal of informed consent, and no follow-up measurements. I.C., informed consent

A total of 175 patients were enrolled and randomized to ipragliflozin or sitagliptin. Of the participants, a total of 15 patients were excluded due to protocol violations (*n* = 8), no baseline measurements (*n* = 2), withdrawal of informed consent (*n* = 3), and no follow-up measurements (*n* = 2). Finally, 160 patients (*n* = 77 and *n* = 83 in the ipragliflozin and sitagliptin groups, respectively) were remained in the analysis (Fig. 1).

### Baseline characteristics and measurements

There were 44 (57.1%) and 44 (53.0%) men in the ipragliflozin and sitagliptin groups, respectively. There were no significant differences in the frequency of alcohol consumption, smoking, comorbidities other than hypertension, and medical history (Table 1). Antidiabetic medications were used by 52 (67.5%) and 56 (67.5%) patients in the ipragliflozin and sitagliptin groups, respectively (*P* = 1.00). The number and class of the medications did not differ between the two groups. For example, the number of participants using pioglitazone was 4 (5.2%) and 3 (3.6%) cases in the ipragliflozin and sitagliptin groups, respectively (*P* = 0.71). Except for systolic blood pressure, there were no significant differences in the clinical and laboratory measurements among the participants (Table 2). Among the other laboratory measurements, serum complement C3 were higher in the ipragliflozin group (Table S3).

**Table 1:** Baseline characteristics of study participants

Parameter	Ipragliflozin (*n* = 77)		Sitagliptin (*n* = 83)		P*
	*n*	%	*n*	%	
Male	44	57.1	44	53.0	0.64
Current smoking	20	26.0	21	25.3	1.00
Current drinking	35	45.5	44	53.0	0.35
Comorbidity					
Diabetic retinopathy	1	1.3	2	2.4	1.00
Diabetic nephropathy	13	16.9	15	18.1	1.00
Dyslipidemia	56	72.7	60	72.3	1.00
Hypertension	62	80.5	50	60.2	0.006
Hyperuricemia	13	16.9	13	15.7	1.00
Liver disease	7	9.1	8	9.6	1.00
Coronary artery disease	2	2.6	3	3.6	1.00
Arteriosclerosis obliterans	4	5.2	3	3.6	0.71
Past medical history					
Malignant neoplasm	4	5.2	1	1.2	0.20
Myocardial infarction	1	1.3	0	0.0	0.48
Stroke	1	1.3	0	0.0	0.48
Heart failure	0	0.0	0	0.0	-

*, *P* < 0.05 indicates the statistical significance based on the Fisher’s exact test for the between-group difference

**Table 2:** Clinical and laboratory measurements at baseline

Parameter	Ipragliflozin			Sitagliptin			P*
	*n*	Mean/median	SD/IQR	*n*	Mean/median	SD/IQR	
Age (year)	77	62	53–67	83	63	54–69	0.19
Height (cm)	77	162.7	8.3	83	161.7	8.4	0.45
Body weight (kg)†	77	69.0	61.8–74.5	83	65.3	59.0–72.8	0.07
Systolic BP (mmHg)	77	138	132–140	83	132	128–138	0.002
Diastolic BP (mmHg)	77	78	76–81	83	76	74–81	0.14
FPG (mmol/L)	62	8.5	7.2–9.8	70	8.6	7.5–10.0	0.34
Insulin (pmol/L)	62	62	39–88	69	58	34–110	0.72
HbA1c (%)	77	7.5	7.1–7.9	82	7.4	7.0–8.0	0.58
GA (%)	77	19.5	17.6–21.0	82	19.6	17.3–22.1	0.61
Lipids							
TC (mg/dL)†	77	223.9	39.8	82	221.3	38.5	0.68
LDL-C (mg/dL)†	58	137.6	33.1	68	131.4	33.4	0.29
HDL-C (mg/dL)	77	46	40–54	82	48	41–61	0.15
TG (mg/dL)	62	161	121–250	70	184	109–239	0.65
Apolipoproteins							
AI (mg/dL)†	77	141.6	23.1	82	146.0	27.7	0.28
AII (mg/dL)	77	29.3	27.1–31.9	82	30.0	26.6–33.3	0.97
B (mg/dL)	77	114	99–139	82	111	94–130	0.54
B48 (µg/L)	77	6.2	3.1–9.7	82	5.6	4.2–10.9	0.58
CII (mg/dL)	77	5.7	4.2–7.6	82	5.1	3.5–6.9	0.13
CIII (mg/dL)	77	11.9	8.9–15.2	82	11.5	8.8–14.3	0.61
E (mg/dL)	77	5.0	4.1–6.1	82	5.0	4.0–5.6	0.57
Cr (mg/dL)	77	0.69	0.57–0.78	82	0.69	0.55–0.83	0.95
CysC (mg/L)	77	0.89	0.78–1.04	81	0.89	0.79–1.01	0.80
Cr-based eGFR (mL/min/1.73 m^2^)†	77	82.4	17.4	82	81.3	18.2	0.72
CysC-based eGFR†	77	81.8	17.1	81	81.8	18.8	0.93
UACR (mg/g Cr)	77	22.9	10.2–71.5	80	13.6	7.3–54.2	0.12

*, *P* < 0.05 indicates the statistical significance based on unpaired t-test or Wilcoxon rank-sum test for the between-group difference

†, Mean and standard deviation (SD) are presented; otherwise, median and interquartile range (IQR) are presented

BP, blood pressure; Cr, creatinine; CysC, cystatin C; FPG, fasting plasma glucose; GA, glycoalbumin; eGFR: estimated glomerular flitration rate, HDL-C, high-density lipoprotein cholesterol; LDL-C, low-density lipoprotein cholesterol; TC, total cholesterol; TG, triglycerides; UACR, urine albumin-to-creatinine ratio

### Glucose metabolism parameters

**Fig. 2:**
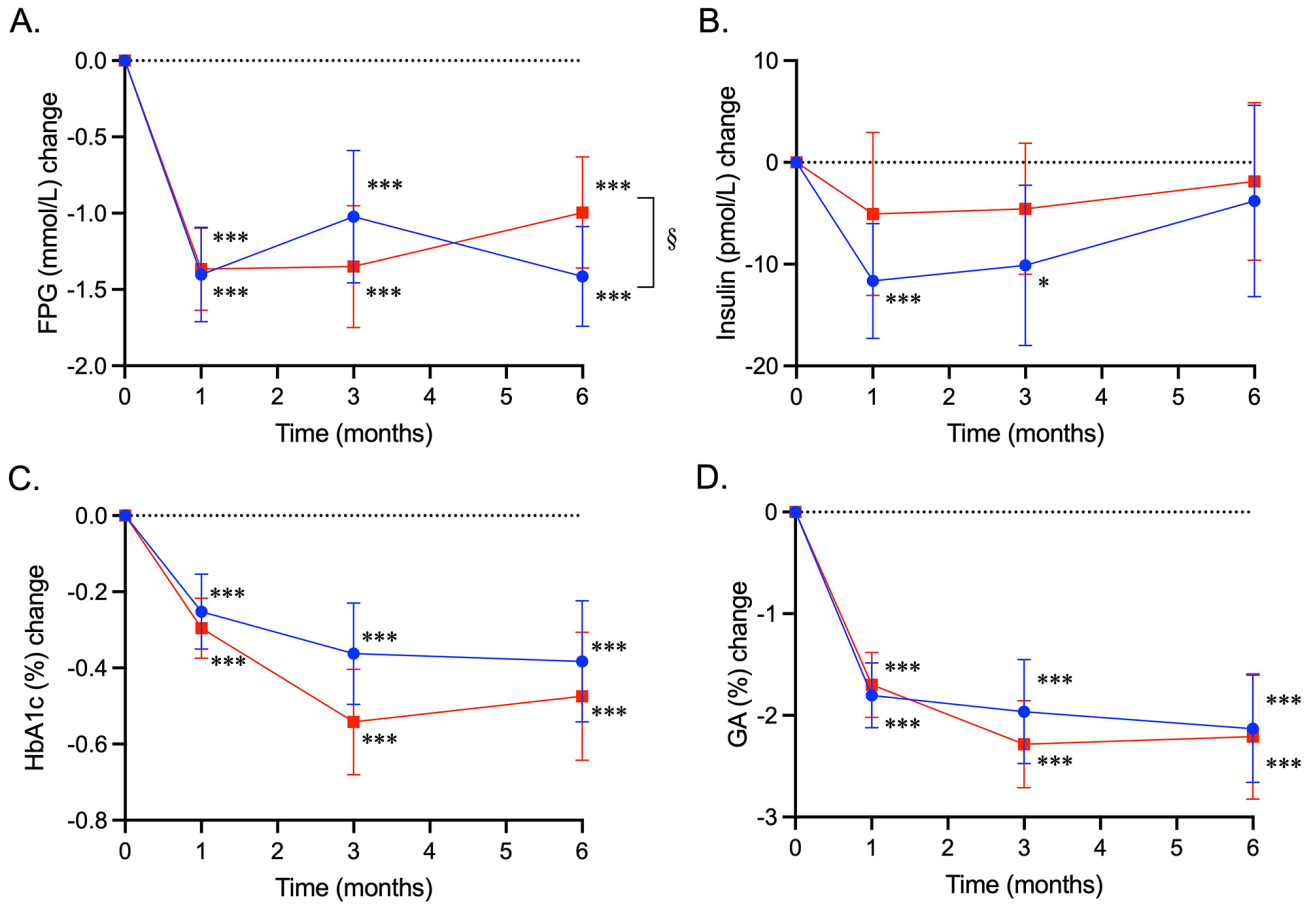
Treatment effects of ipragliflozin and sitagliptin on glucose metabolism parameters. Changes from baseline in fasting plasma glucose (FPG, mmol/L) (**A**), serum insulin (pmol/L) (**B**), HbA1c (%) (**C**), and glycoalbumin (GA, %) (**D**) in the ipragliflozin and sitagliptin groups are shown as closed circles with blue lines and closed squares with red lines, respectively. Data are expressed as mean ± 95% confidence interval of the mean. *, *P* < 0.05; ***, *P* < 0.001 vs. baseline in each group in the mixed-effects model analysis. §, *P* < 0.05 for interaction during the overall study period, vs. the sitagliptin group in the mixed-effects model analysis

Changes in glucose metabolism parameters from baseline are shown in Fig. 2; Table 3. FPG, HbA1c, and GA were significantly decreased by the treatments to almost the same extent in both groups (Fig. 2A and C, and 2D). Serum insulin decreased at 1 and 3 months in the ipragliflozin group (Fig. 2B).

**Table 3:** Overall changes from baseline in glucose metabolism parameters and serum lipids and apolipoproteins

Parameter	Adjusted overall change (95% CI)*		*P*†	
	Ipragliflozin	Sitagliptin	Difference	Interaction
Glucose metabolism				
FPG (mmol/L)	−1.28 (− 1.56; −1.00)	−1.24 (− 1.51; −0.96)	0.83	0.04
Insulin (pmol/L)	−8.51 (− 14.7; −2.34)	−3.83 (− 9.39; 1.74)	0.27	0.75
HbA1c (%)	−0.33 (− 0.45; −0.22)	−0.44 (− 0.55; −0.33)	0.19	0.14
GA (%)	−1.97 (− 2.35; −1.58)	−2.07 (− 2.44; −1.69)	0.72	0.29
Lipids				
TC (mg/dL)	1.7 (− 3.6; 6.9)	−1.8 (− 6.8; 3.3)	0.36	0.84
LDL-C (mg/dL)	1.2 (− 3.1; 5.6)	6.6 (1.6; 11.6)	0.12	0.40
HDL-C (mg/dL)	2.3 (0.9; 3.6)	0.0 (− 1.2; 1.3)	0.02	0.59
TG (mg/dL)	−12.7 (− 30.3; 4.9)	−23.1 (− 40.7; −5.4)	0.42	0.92
Apolipoproteins				
AI (mg/dL)	2.26 (− 0.89; 5.40)	−1.68 (− 4.23; 0.87)	0.06	0.86
AII (mg/dL)	−0.51 (− 1.17; 0.16)	−0.18 (− 0.74; 0.39)	0.46	0.26
B (mg/dL)	−0.69 (− 3.90; 2.52)	−1.96 (− 4.74; 0.81)	0.55	0.59
B48 (µg/dL)	0.45 (− 0.60; 1.49)	−1.39 (− 2.16; −0.61)	0.006	0.11
CII (mg/dL)	−0.21 (− 0.51; 0.09)	−0.40 (− 0.64; −0.17)	0.31	0.71
CIII (mg/dL)	−0.07 (− 0.85; 0.72)	−0.86 (− 1.38; −0.34)	0.10	0.51
E (mg/dL)	−0.30 (− 0.56; −0.05)	−0.09 (− 0.30; 0.11)	0.21	0.91

Follow-up measurements were done at 1, 3, and 6 months

*, Based on a mixed-model repeated-measures analysis adjusting for sex, age, and baseline value of a parameter of interest

†, *P* < 0.05 indicates the statistical significance for the between-treatment difference and treatment-month interaction

CI, confidence interval; FPG, fasting plasma glucose; GA, glycoalbumin; HDL-C, high-density lipoprotein cholesterol; LDL-C, low-density lipoprotein cholesterol; TC, total cholesterol; TG, triglycerides

### Serum lipids and apolipoproteins

**Fig. 3:**
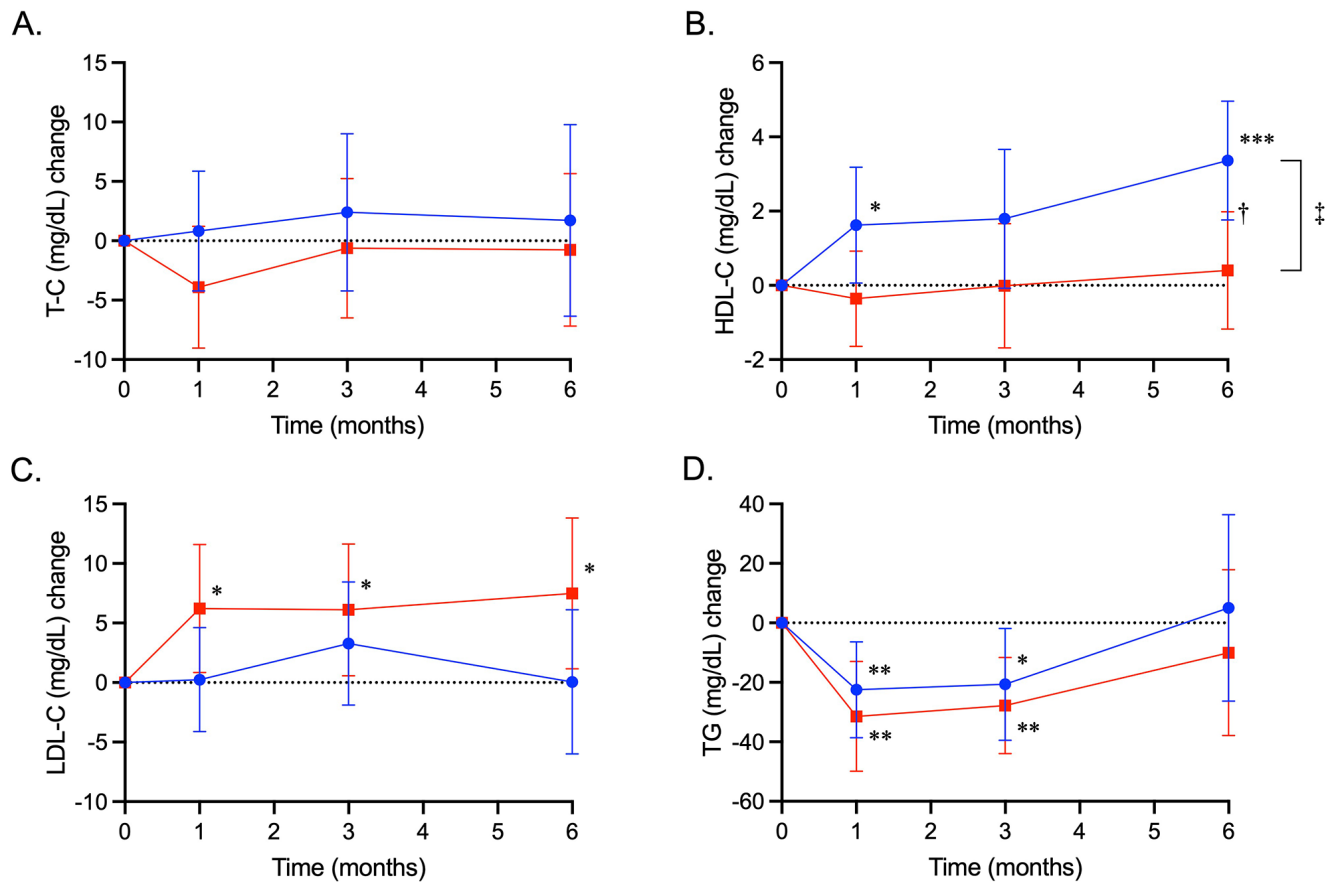
Treatment effects of ipragliflozin and sitagliptin on serum lipids. Changes from baseline in serum total cholesterol (TC, mg/dL) (**A**), high-density lipoprotein cholesterol (HDL-C, mg/dL) (**B**), low-density lipoprotein cholesterol (LDL-C, mg/dL) (**C**), and triglycerides (TG, mg/dL) (**D**) in the ipragliflozin and sitagliptin groups are shown as closed circles with blue lines and closed squares with red lines, respectively. Data are expressed as mean ± 95% confidence interval of the mean. *, *P* < 0.05; **, *P* < 0.01; ***, *P* < 0.001 vs. baseline in each group; †, *P* < 0.05 for difference at the follow up visit; ‡, *P* < 0.05 for difference during the overall study period, vs. the sitagliptin group in the mixed-effects model analysis

**Fig. 4:**
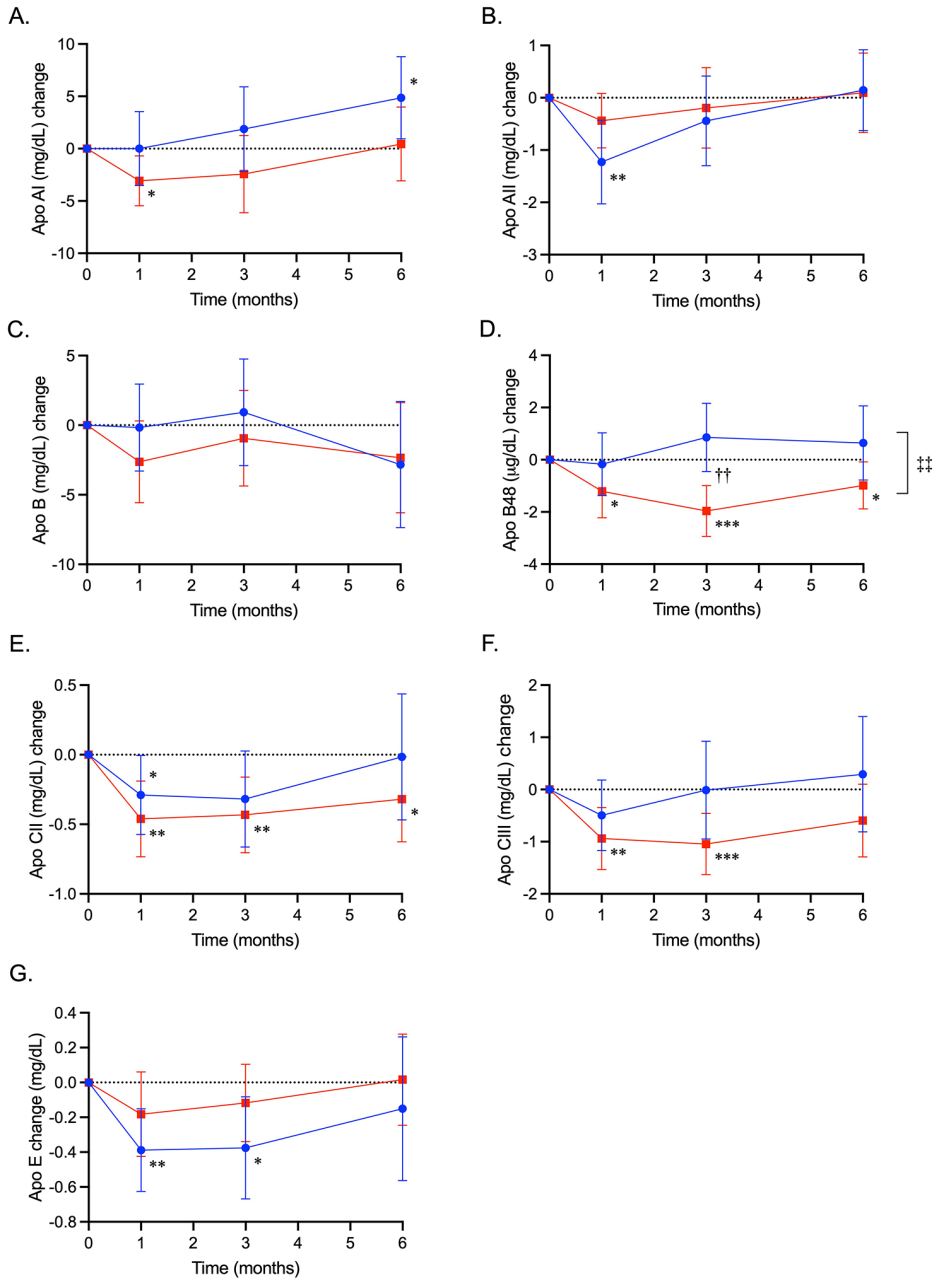
Treatment effects of ipragliflozin and sitagliptin on serum apolipoproteins. Changes from baseline in serum apolipoprotein (Apo) AI (mg/dL) (**A**), AII (mg/dL) (**B**), B (mg/dL) (**C**), B48 (µg/dL) (**D**), CII (mg/dL) (E), CIII (mg/dL) (F), and E (mg/dL) (G) in the ipragliflozin and sitagliptin groups are shown as closed circles with blue lines and closed squares with red lines, respectively. Data are expressed as mean ± 95% confidence interval of the mean. *, *P* < 0.05; **, *P* < 0.01, ***, *P* < 0.001 vs. baseline in each group; ††, *P* < 0.01 for difference at the follow up visit; ‡‡, *P* < 0.01 for difference during the overall study period, vs. the sitagliptin group in the mixed-effects model analysis

Changes in serum lipids and apolipoproteins from baseline are shown in Figs. 3 and 4; Table 3. Although TC did not change in either group (Fig. 3A), LDL-C increased consistently in the sitagliptin group; TG decreased transiently in both groups (Fig. 3C and D); and HDL-C increased fairly consistently in the ipragliflozin group (Fig. 3B). A significant between-treatment difference was observed only for HDL-C (Table 3).

Apo AI increased gradually and apo AII decreased transiently in the ipragliflozin group (Fig. 4A and B). Although apo B did not change in either group, apo B48 decreased consistently in the sitagliptin group (Fig. 4C and D). Apo CII decreased consistently and apo CIII decreased almost consistently in the sitagliptin group (Fig. 4E and F). Apo E decreased transiently in the ipragliflozin group (Fig. 4G). Consequently, overall reductions in apo E were observed in the ipragliflozin group, and in apo B48, CII, and CIII in the sitagliptin group (Table 3). A significant between-treatment difference was observed for apo B48 and a near-significant difference for apo A1 (Fig. 4D; Table 3).

### Body weight, blood pressure, and renal function

Changes from baseline in the secondary outcome measures other than glucose metabolism parameters are shown in Table 4. Body weight and blood pressure decreased significantly in the ipragliflozin group but not in the sitagliptin group, resulting in overall significant between-treatment differences. In the ipragliflozin group, the reduction in body weight was greater in the later months of follow-up (*P* < 10^− 3^ for interaction). Regarding renal function parameters, eGFR decreased consistently in both groups during the treatment period, although the decrease in Cr-based eGFR was less pronounced in the ipragliflozin group. The overall reduction in UACR was significant in the ipragliflozin group, but there was no between-treatment difference.

**Table 4:** Changes from baseline in secondary outcome measures other than glucose metabolism parameters

Parameter	Month	Adjusted mean change (95% CI)*		*P*†	
		Ipragliflozin	Sitagliptin	Difference	Interaction
Body weight (kg)	1	−0.7 (− 1.0; −0.5)	−0.2 (− 0.5; 0.0)	0.009	
	3	−1.2 (− 1.7; −0.8)	−0.1 (− 0.4; 0.2)	< 10^− 4^	
	6	−1.8 (− 2.3; −1.4)	−0.3 (− 0.7; −0.0)	< 10^− 6^	
	Overall	−1.4 (− 1.7; −1.0)	−0.2 (− 0.5; 0.0)	< 10^− 5^	< 10^− 3^
Systolic BP (mmHg)	1	−3.9 (− 5.3; −2.6)	−1.7 (− 3.4; −0.0)	0.04	
	3	−4.1 (− 6.0; −2.3)	0.8 (− 1.0; 2.7)	< 10^− 3^	
	6	−3.8 (− 5.5; −2.1)	0.7 (− 1.3; 2.7)	0.001	
	Overall	−3.9 (− 5.2; −2.7)	−0.1 (− 1.5; 1.4)	< 10^− 4^	0.08
Diastolic BP (mmHg)	1	−3.2 (− 4.7; −1.8)	−2.1 (− 3.4; −0.8)	0.25	
	3	−3.0 (− 4.7; −1.4)	−0.4 (− 1.9; 1.1)	0.02	
	6	−3.4 (− 4.6; −2.2)	−0.7 (− 2.3; 0.8)	0.008	
	Overall	−3.2 (− 4.4; −2.0)	−1.1 (− 2.1; −0.1)	0.009	0.28
Cr-based eGFR (mL/min/1.73 m^2^)	1	−1.8 (− 4.6; 1.0)	−2.6 (− 4.4; −0.7)	0.65	
	3	−1.0 (− 3.2; 1.2)	−3.8 (− 5.6; −2.1)	0.05	
	6	−2.4 (− 5.2; 0.5)	−5.1 (− 7.0; −3.3)	0.11	
	Overall	−1.7 (− 3.8; 0.4)	−3.9 (− 5.3; −2.5)	0.10	0.45
CysC-based eGFR (mL/min/1.73 m^2^)	1	−3.3 (− 5.5; −1.1)	−1.3 (− 2.8; 0.3)	0.13	
	3	−2.2 (− 4.4; −0.1)	−2.4 (− 4.0; −0.8)	0.91	
	6	−3.4 (− 5.8; −1.0)	−2.9 (− 4.7; −1.1)	0.76	
	Overall	−3.0 (− 4.8; −1.2)	−2.2 (− 3.5; −0.9)	0.49	0.28
UACR (mg/g Cr)‡	1	−0.12 (− 0.33; 0.08)	−0.15 (− 0.38; 0.08)	0.87	
	3	−0.16 (− 0.41; 0.09)	−0.17 (− 0.44; 0.10)	0.95	
	6	−0.25 (− 0.48; −0.02)	−0.13 (− 0.38; 0.12)	0.52	
	Overall	−0.18 (− 0.34; −0.01)	−0.15 (− 0.34; 0.04)	0.84	0.76

*, Based on a mixed mixed-model repeated-measures analysis adjusting for sex, age, and baseline value of a parameter of interest

†, *P* < 0.05 indicates the statistical significance for the between-treatment difference and treatment-month interaction

‡, In the natural log-scale

CI, confidence interval; BP, blood pressure; Cr, creatinine; CysC, cystatin C; eGFR, estimated glomerular filtration rate; UACR, urine albumin-to-creatinine ratio

### Other laboratory measurements

Overall changes in other laboratory measurements during the treatment period are summarized in Table S4. Between-treatment differences were observed in blood cell counts except for white blood cells and platelets, serum liver function parameters (AST, ALT, GGT, and LDH), uric acid, leptin, and ketones. Most of the differences were induced by ipragliflozin treatment, such as increases in blood cell counts and ketones, and decreases in liver function parameters, uric acid, and leptin.

### Adverse events

The analysis of adverse events included 171 patients who received actual treatment with each drug; 80 and 91 patients with ipragliflozin and sitagliptin, respectively. Reported adverse events are summarized in Table S5. The number of patients reporting at least one adverse event was 20 (25.0%) in the ipragliflozin group and 7 (7.7%) in the sitagliptin group. Skin lesions and elevated blood ketones were reported only in the ipragliflozin group. Two patients in the ipragliflozin group discontinued treatment due to the occurrence of rash or cholelithiasis/cholecystitis.

## Discussion

In this multicenter, open-label, randomized, controlled trial, we found that although both ipragliflozin and sitagliptin reduced glucose metabolism parameters to a similar extent, the effects on serum lipid and apolipoprotein profiles were completely different. Ipragliflozin increased HDL-C and decreased apo E, whereas sitagliptin decreased TG, apo B48, CII, and CIII but increased LDL-C. Ipragliflozin was more likely to improve cardiovascular disease risk factors, i.e., HDL-C, body weight, blood pressure, and uric acid, compared with sitagliptin.

To date, three randomized controlled trials have compared ipragliflozin and sitagliptin head-to-head in patients with type 2 diabetes [23–25]. Contrary to the present finding, these studies reported greater reductions in FPG with ipragliflozin than with sitagliptin at 12 [23], 24 [24], and 52 weeks [25] of treatment, whereas reductions in HbA1c did not show a measurable difference between the two treatments [23–25]. These findings may indicate that sitagliptin primarily act on the improvement of postprandial blood glucose levels compared to ipragliflozin. However, under the conditions of the present study, both ipragliflozin and sitagliptin reduced FPG and HbA1c to a similar extent. Furthermore, GA, which reflects postprandial blood glucose levels more accurately than HbA1c, was similarly decreased by both drugs. We do not have a direct explanation for the present findings that differ from previous observations, but the discrepancy in FPG may be due to differences in study design regarding the use of other antidiabetic drugs. The present study allowed adjustment of existing antidiabetic drugs after the first 3 months of treatment, whereas the previous studies did not allow such adjustment. However, the reductions in FPG did not differ between the two treatments even at 1 month and 3 months when adjustment for antidiabetic drugs was not allowed. The previous studies [23, 24] and the present study showed that ipragliflozin reduced body weight more, accompanied by increased levels of ketone bodies, as compared with sitagliptin. This could be due to increased fat oxidation as a result of accelerated urinary glucose excretion caused by the SGLT2 inhibitory effect of ipragliflozin. In addition, it has been suggested that body weight loss with ipragliflozin can be partly explained by a reduction in extracellular water [26], as SGLT2 inhibitors have an osmotic diuretic effect with urinary excretion of sodium accompanied by glucose [27]. This consideration may be supported by the reductions in blood pressure and serum uric acid, and increases in red blood cell count and hematocrit in the ipragliflozin group in the present study.

The present study showed that ipragliflozin and sitagliptin had different effects on lipid and apolipoprotein profiles despite similar hypoglycemic effects. Ipragliflozin increased HDL-C without affecting TC and LDL-C, whereas sitagliptin decreased TG but increased LDL-C without affecting TC and HDL-C. In a meta-analysis by Li, et al. [16]. , SGLT2 inhibitors increased TC, LDL-C, and HDL-C and decreased TG. However, the effects of SGLT2 inhibitors on TC and TG were not consistent across studies. On the other hand, an increase in HDL-C with SGLT2 inhibitors was consistently found in meta-analyses [28, 29]. Several possible mechanisms have been proposed for the HDL-C increase with SGLT2 inhibitors; (1) increased lipolysis of triglycerides, (2) decreased CETP activity, and (3) de novo production of nascent HDL [30, 31]. According to the finding that apo A1 was gradually increased by ipragliflozin in the present study, the HDL increase by SGLT2 inhibitors is more likely explained by de novo synthesis of apo AI and subsequent production of nascent HDL. Notably, although there was no difference between treatments, apo E was significantly reduced in the ipragliflozin group. The liver is a major source of apo E [32], and elevated plasma apo E levels have been reported in patients with non-alcoholic fatty liver disease (NAFLD) regardless of apo E genotype [33]. Since ipragliflozin has been shown to improve hepatic steatosis [34], and serum liver enzymes were reduced in the present study, circulating apo E may have been reduced as hepatic steatosis was improved. On the other hand, sitagliptin reduced TG and apo B48 without affecting HDL-C and apo AI. These results suggest that the effect of sitagliptin on triglyceride metabolism can be explained by an inhibition of lipoprotein production from the small intestine, as manifested by decreases in apo B48 and CII, and not by a lipolytic activity associated with an increase in HDL-C. These findings are consistent with previous reports that sitagliptin suppressed postprandial triglyceride elevation [17] and reduced fasting apo B48 in addition to CII [22]. In addition, a single-arm, observational study of sitagliptin treatment in drug naïve patients with type 2 diabetes suggested that sitagliptin might improve atherogenic lipids in a glycemic efficacy dependent manner [35]. However, the present study would not be able to address such a hypothesis based on patients with a baseline HbA1c of 10.2%, which was much higher than the baseline HbA1c of 7.4% in the sitagliptin group.

SGLT2 inhibitors slow the decline in eGFR in patients with type 2 diabetes, even with a transient reduction in eGFR early in treatment [36]. DDP-4 inhibitors have been reported to be associated with a continuous decline in eGFR [37]. The present study showed that both ipragliflozin and sitagliptin decreased eGFR, particularly CysC-based eGFR, to a similar extent up to 6 months of treatment. In contrast to eGFR reduction, both SGLT2 and DDP-4 inhibitors are protective against worsening of albuminuria and may even reduce UACR [36–38]. In the present study, we could show a statistically significant UACR reduction with ipragliflozin treatment, but not with sitagliptin treatment, although the between-treatment difference was not appreciable. The findings are compatible with the recent observation in the network meta-analysis on SGLT2 inhibitors and DDP-4 inhibitors [39].

The present study has several limitations. First, it was an open-label study. Second, the number of cases was insufficient to detect between-treatment differences in changes in apolipoproteins when within-treatment differences were detected. Because more adverse events were observed in the ipragliflozin group, the smaller number of cases in this group may have influenced the study results. Third, dietary control was not strictly prescribed during the follow-up period. Fourth, the study protocol allowed for the addition, dose titration, or discontinuation of oral antidiabetic drugs other than SGLT2 inhibitors, DPP4 inhibitors, and GLP-1 receptor agonists after 3 months of treatment. In fact, changes in drug regimens were only made within the insulin sensitizer category. For example, metformin was started in four cases in the ipragliflozin group and pioglitazone in one case in the sitagliptin group. In addition, metformin and/or pioglitazone were discontinued in two cases in the sitagliptin group. However, this may have allowed us to evaluate the effects of ipragliflozin and sitagliptin on lipid and apolipoprotein profiles independent of glycemic levels. Fifth, we did not have detailed information on antihypertensive drugs while the prevalence of hypertension was higher in the ipragliflozin group. In fact, the participants using antihypertensive drugs were more frequent in the ipragliflozin group (*n* = 54, 70.1%) than in the sitagliptin group (*n* = 43, 51.8%) (*P* = 0.02). Some antihypertensive drugs have a neutral or beneficial effect on the lipid profile, while others have an adverse effect. Different use of antihypertensive drugs may influence the lipid profile. Finally, although the intent-to-treat (ITT) analysis is a principle in the randomized trial, the present study excluded patients with no follow-up measurements and those who did not follow to the protocol, necessarily causing the so-called attrition bias. The present study was exploratory, and the observed findings should be validated in the ITT analysis in larger randomized trials in the future. Further investigations, including animal or ex vivo studies, are also needed to fully understand the pharmacological effects of these drugs on lipid metabolism.

## Conclusions

In summary, although ipragliflozin and sitagliptin showed similar effects on glycemic parameters in the present study, the effects of these drugs on lipid and apolipoprotein profiles were different. Both drugs have favorable effects on the profiles, i.e., increased HDL-C and decreased apo E with ipragliflozin and decreased TG, apo B48, CII, and CIII with sitagliptin. Ipragliflozin is more likely than sitagliptin to contribute to plaque reduction by improving cardiovascular disease risk factors, including HDL-C, body weight, blood pressure, and uric acid. These findings in the present study suggest a strong need for further large clinical trials to validate the protective effects of ipragliflozin on cardiovascular outcomes.

### Electronic supplementary material

Below is the link to the electronic supplementary material.

Supplementary Material 1

## Data Availability

Some or all datasets generated and/or analyzed during the current study are not publicly available but are available from the corresponding author on reasonable request.

## Abbreviations

SGLT2: Sodium-glucose cotransporter 2
DPP4: Dipeptidyl peptidase-4
SUCRE: Sodium-GlUose Co-transporteR 2 inhibitor, Ipragliflozin, Suglat®, Effect on Lipid and Glucose Metabolism
UMIN: University hospital Medical Information Network
LDL-C: Low-density lipoprotein cholesterol
HDL-C: High-density lipoprotein cholestero
TG: Triglycerides
HbA1c: Glycohemoglobin
Cr: Creatinine
NYHA: New York Heart Association
GLP-1: Glucagon-like peptide-1
Apo: Apolipoprotein
FPG: Fasting plasma glucose
GA: Glycoalbumin
eGFR: Estimated glomerular filtration rate
UACR: Urine albumin-to-Cr ratio
CysC: Cystatin C
SD: Standard deviation
IQR: Interquartile range
LDH: Lactate dehydrogenase
AST: Aspartate aminotransferase
ALT: Alanine aminotransferase
GGT: Gamma-glutamyl transferase
NAFLD: Non-alcoholic fatty liver disease
ITT: Intent-to-treat
