# Supplementary material for: Ipragliflozin and sitagliptin differentially affect lipid and apolipoprotein profiles in type 2 diabetes: the SUCRE study
Source: Cardiovasc Diabetol. 2024 Feb 8;23:56. doi: 10.1186/s12933-024-02149-7 (PMC10854175; doi:10.1186/s12933-024-02149-7)
Supplement: Supplementary file 1 — Supplementary Material 1 [file 12933_2024_2149_MOESM1_ESM.docx]

**Table S1. Methods and resources used for the SUCRE study**

| Parameter | Method | Resource | Source | Identifier |
| --- | --- | --- | --- | --- |
| Glucose | Hexokinase UV method | CicaLiquid Glu J | Kanto Chemical | L360K |
| HbA1c | Chemiluminescence enzyme immunoassay | MetaboLead HbA1c | Mirnaris Medical | 473-07071, 470-07081 |
| Insulin | Chemiluminescence enzyme immunoassay | LUMIPULSE Presto Insulin | Fujirebio | 291290 |
| GA | Visible absorption spectrometry enzymatic method | Lucica GA-L | Asahikasei pharma | L360K |
| TC | Cholesterol dehydrogenase UV method | Cholestest CHO | Sekisui Medical | 318061, 318030 |
| TG | Enzymatic method  (GK-GPO free glycerol blank) | PureautoS TG-N | Sekisui Medical | 225819, 225826 |
| HDL-C | Direct method | Cholestest N HDL | Sekisui Medical | 290268, 290343 |
| Apo AI/AII/B/CII/CIII/E | Turbidimetric immunoassay | Apo AI/AII/B/CII/CIII/E Auto N | Sekisui Medical | 241765/241789/241819/241819/241833/241871/241871 |
| Apo B48 | Chemiluminescence enzyme immunoassay | Apo B48 CLEA | Fujirebio | 261040 |
| Cr | Visible absorption spectrometry enzymatic method | Determiner L CRE | Minaris Medical | 26272-5, 26273-2 |
| CysC | Gold colloidal agglomeration method | Nescoat GC Cystatin C (Nm) | Alfresa Pharma | 4987274080333 |
| Urinary albumin | Turbidimetric immunoassay | Auto Wako microalbumin | Fujifilm Wako Chemicals | 467-75201 |
| AST/ALT/GGT/LDH/CPK | JSCC reference method | CicaLiquid AST/ALT/γGT J/ LDH J/CK | Kanto Chemical | 78282/75706,75458/75786/78286/77798,77799 |
| UA | Enzymatic method  (Uricase POD method) | PureautoS UA | Sekisui Medical | 219511, 218491 |
| Na/K/Cl | Electrode method | Na/K/Cl measurement kit | A & T | N/A |
| Leptin | RIA antibody method | Human Leptin RIA kit | Millipore Corporation | HL-81K |
| Non-cholesterol sterols | Gas chromatography | Inertcap 5MS | GL Sciences | N/A |
| Cholestanol | Gas chromatography | Capillary column HP-5 | Agilent Technologies | N/A |
| CH50 | Mayer method, relative turbidity method | Auto CH50-L Seiken II | Denka | 400482 |
| C3/C4 | Turbidimetric immunoassay | N-assay TIA C3-SH/C4-SH | Nittobo Medical | 14351154,14351254,9490533/14352154,14352254,9490533 |
| Fatty acids | Gas chromatography | TC-70 | GL Sciences | N/A |
| Ketones | Chemiluminescence enzyme immunoassay | 3HB-L reagent kainos | Kainos | CL-5521, CL-5522 |

Cr, creatinine; CysC, cystatin C; GA, glycoalbumin; GGT, gamma-glutamyl transferase; HDL-C, high-density lipoprotein cholesterol; LDH, lactate dehydrogenase; N/A, not available; TC, total cholesterol; TG, triglycerides; UA, uric acid.

**Table S2. Number of patients analyzed at follow-up time points**

| Measurement parameter | Month | Ipragliflozin | Sitagliptin |
| --- | --- | --- | --- |
| Body weight | 1 | 38 | 44 |
|  | 3 | 77 | 82 |
|  | 6 | 76 | 83 |
| Blood pressure | 1 | 77 | 81 |
|  | 3 | 77 | 82 |
|  | 6 | 76 | 83 |
| FPG | 1 | 52 | 57 |
|  | 3 | 53 | 58 |
|  | 6 | 54 | 56 |
| Insulin | 1 | 52 | 56 |
|  | 3 | 53 | 57 |
|  | 6 | 54 | 55 |
| HbA1c and GA | 1 | 74 | 80 |
|  | 3 | 77 | 80 |
|  | 6 | 76 | 80 |
| TC and HDL-C | 1 | 74 | 80 |
|  | 3 | 77 | 80 |
|  | 6 | 76 | 80 |
| LDL-C | 1 | 50 | 54 |
|  | 3 | 51 | 56 |
|  | 6 | 50 | 51 |
| TG | 1 | 52 | 57 |
|  | 3 | 53 | 58 |
|  | 6 | 54 | 56 |
| Apolipoproteins, Cr, and CysC | 1 | 74 | 80^*^ |
|  | 3 | 77† | 80^*^ |
|  | 6 | 76 | 80^*^ |
| Blood cell counts | 1 | 73 | 80 |
|  | 3 | 77 | 80 |
|  | 6 | 76 | 80 |
| Serum enzymes and uric acid | 1 | 74 | 80^*^ |
|  | 3 | 77 | 80^*^ |
|  | 6 | 76 | 80^*^ |
| Serum electrolytes, complements, leptin, fatty acids, and ketones | 1 | 74 | 79 |
|  | 3 | 77^†^ | 79 |
|  | 6 | 76^‡^ | 79 |
| Serum non-cholesterol sterols | 1 | 74 | 81 |
|  | 3 | 76 | 81 |
|  | 6 | 75 | 80 |
| Urinary albumin | 1 | 74 | 78 |
|  | 3 | 76 | 78 |
|  | 6 | 76 | 78 |

*, n = 79 for apolipoproteins CII, CIII and E, CysC, and LDH.

†, n = 76 for apolipoprotein B48 and ketones.

‡, n = 75 for leptin.

Cr, creatinine; CysC, cystatin C; FPG, fasting plasma glucose; GA, glycoalbumin; HDL-C, high-density lipoprotein cholesterol; LDL-C, low-density lipoprotein cholesterol; TC, total cholesterol; TG, triglycerides.

**Table S3. Laboratory measurements other than primary and secondary outcomes at baseline**

| Parameter | Ipragliflozin | | |  | Sitagliptin | | | *P** |
| --- | --- | --- | --- | --- | --- | --- | --- | --- |
|  | n | Mean/median | SD/IQR |  | n | Mean/median | SD/IQR |  |
| Blood cell counts |  |  |  |  |  |  |  |  |
| White blood cell (×10^2^/μL) | 77 | 64 | 52–72 |  | 82 | 60 | 52–69 | 0.24 |
| Red blood cell (×10^4^/μL) | 77 | 478 | 454–505 |  | 82 | 481 | 455–515 | 0.82 |
| Hemoglobin (g/dL) | 77 | 14.4 | 13.6–15.5 |  | 82 | 14.8 | 13.7–15.5 | 0.67 |
| Hematocrit (%) | 77 | 43.9 | 41.6–46.6 |  | 82 | 43.9 | 42.0–46.8 | 0.64 |
| Platelet (×10^4^/μL)† | 77 | 25.5 | 5.0 |  | 82 | 24.5 | 5.5 | 0.22 |
| Serum enzymes |  |  |  |  |  |  |  |  |
| AST (U/L) | 77 | 23 | 18–31 |  | 82 | 21 | 17–30 | 0.46 |
| ALT (U/L) | 77 | 26 | 18–50 |  | 82 | 25 | 15–42 | 0.21 |
| GGT (U/L) | 77 | 39 | 25–63 |  | 82 | 33 | 22–55 | 0.21 |
| LDH (U/L) | 77 | 182 | 160–218 |  | 81 | 178 | 156–200 | 0.12 |
| CPK (U/L) | 77 | 89 | 64–136 |  | 82 | 83 | 65–115 | 0.26 |
| Serum uric acid (mg/dL) | 77 | 5.5 | 4.5–6.2 |  | 82 | 5.3 | 4.6–6.0 | 0.74 |
| Serum electrolytes |  |  |  |  |  |  |  |  |
| Sodium (mEq/L) | 77 | 140 | 139–141 |  | 81 | 140 | 139–141 | 0.26 |
| Chloride (mEq/L)† | 77 | 102.8 | 2.6 |  | 81 | 103.4 | 2.4 | 0.09 |
| Potassium (mEq/L) | 77 | 4.3 | 4.0–4.5 |  | 81 | 4.3 | 4.1–4.6 | 0.31 |
| Serum complements |  |  |  |  |  |  |  |  |
| Complement titer  (CH50/mL) | 77 | 46.9 | 42.7–51.4 |  | 81 | 44.7 | 40.4–48.9 | 0.04 |
| C3 (mg/dL)† | 77 | 131.1 | 20.9 |  | 81 | 121.2 | 19.6 | 0.002 |
| C4 (mg/dL) | 77 | 28 | 23–35 |  | 81 | 27 | 24–34 | 0.39 |
| Serum leptin (ng/mL) | 77 | 12.7 | 8.6–19.6 |  | 81 | 13.3 | 7.4–22.6 | 0.89 |
| Serum non-cholesterol sterols |  |  |  |  |  |  |  |  |
| Lathosterol (μg/mL) | 77 | 3.1 | 2.4–4.5 |  | 83 | 2.8 | 2.1–4.0 | 0.09 |
| Campesterol (μg/mL) | 77 | 4.6 | 3.2–5.5 |  | 83 | 4.3 | 3.2–6.2 | 0.997 |
| Sitosterol (μg/mL) | 77 | 2.2 | 1.6–3.1 |  | 83 | 2.2 | 1.5–3.1 | 0.999 |
| Cholestanol (μg/mL) | 77 | 2.6 | 2.1–3.1 |  | 83 | 2.5 | 2.1–3.2 | 0.62 |
| Serum fatty acids |  |  |  |  |  |  |  |  |
| Dihomo-γ-linolenic acid  (μg/mL) | 77 | 49.2 | 41.8–64.3 |  | 81 | 47.8 | 39.0–56.2 | 0.22 |
| AA (μg/mL) | 77 | 211.4 | 179.1–254.9 |  | 81 | 214.8 | 177.0–250.8 | 0.85 |
| EPA (μg/mL) | 77 | 58.1 | 40.5–92.9 |  | 81 | 64.5 | 47.6–96.3 | 0.35 |
| DHA (μg/mL) | 77 | 150.7 | 117.8–192.9 |  | 81 | 155.0 | 110.6–193.1 | 0.88 |
| EPA/AA ratio | 77 | 0.28 | 0.19–0.42 |  | 81 | 0.30 | 0.21–0.43 | 0.33 |
| Serum ketones |  |  |  |  |  |  |  |  |
| Acetoacetic acid (μmoL/L) | 77 | 17 | 10–27 |  | 81 | 16 | 11–22 | 0.51 |
| 3-OH butyric acid  (μmoL/L) | 77 | 43 | 24–85 |  | 81 | 37 | 23–55 | 0.23 |
| Total ketones (μmoL/L) | 77 | 62 | 35–112 |  | 81 | 52 | 34–71 | 0.22 |

*, *P* < 0.05 indicates the statistical significance based on unpaired *t*-test or Wilcoxon rank-sum test for the between-group difference.

†, Mean and standard deviation (SD) are presented; otherwise, presented are median and interquartile range (IQR).

AA, arachidonic acid; AST, aspartate aminotransferase; ALT alanine aminotransferase; CPK, creatine phosphokinase; DHA, docosahexaenoic acid; EPA, eicosapentaenoic acid; GGT, gamma-glutamyl transferase; LDH, lactate dehydrogenase.

**Table S4. Overall changes from baseline in other laboratory measurements**

| Parameter | Adjusted overall change (95% CI)* | |  | *P*† | |
| --- | --- | --- | --- | --- | --- |
|  | Ipragliflozin | Sitagliptin |  | Difference | Interaction |
| Blood cell counts |  |  |  |  |  |
| White blood cell (per μL) | −54 (−279; 171) | 138 (−118; 394) |  | 0.25 | 0.21 |
| Red blood cell (×10^4^/μL) | 20.1 (14.9; 25.4) | −3.61 (−7.25; 0.03) |  | <10^−12^ | 0.04 |
| Hemoglobin (g/dL) | 0.58 (0.41; 0.75) | −0.08 (−0.19; 0.03) |  | <10^−9^ | 0.30 |
| Hematocrit (%) | 1.94 (1.48; 2.41) | −0.16 (−0.48; 0.16) |  | <10^−12^ | 0.45 |
| Platelet (×10^4^/μL) | 0.30 (−0.35; 0.96) | −0.17 (−0.91; 0.57) |  | 0.36 | 0.12 |
| Serum enzymes |  |  |  |  |  |
| AST (U/L) | −2.9 (−4.5; −1.4) | 0.7 (−1.7; 3.2) |  | 0.01 | 0.29 |
| ALT (U/L) | −5.5 (−7.9; −3.2) | 0.6 (−3.3; 4.4) |  | 0.009 | 0.11 |
| GGT (U/L) | −8.4 (−12.4; −4.4) | 1.9 (−4.4; 8.3) |  | 0.006 | 0.73 |
| LDH (U/L) | −9.9 (−14.7; −5.2) | −2.2 (−6.7; 2.4) |  | 0.02 | 0.006 |
| CPK (U/L) | 8.3 (−24.3; 40.9) | 6.4 (−6.4; 19.1) |  | 0.91 | 0.48 |
| Serum uric acid (mg/dL) | −0.41 (−0.56; −0.25) | 0.30 (0.15; 0.46) |  | <10^−9^ | 0.09 |
| Serum electrolytes |  |  |  |  |  |
| Sodium (mEq/L) | 0.55 (0.27; 0.82) | 0.17 (−0.14; 0.49) |  | 0.08 | 0.56 |
| Chloride (mEq/L) | 0.59 (0.26; 0.93) | 0.41 (0.08; 0.73) |  | 0.44 | 0.30 |
| Potassium (mEq/L) | −0.00 (−0.07; 0.07) | −0.02 (−0.07; 0.03) |  | 0.68 | 0.03 |
| Serum complements |  |  |  |  |  |
| Titer (CH50/mL) | −0.41 (−1.44; 0.61) | −1.01 (−2.02; 0.01) |  | 0.41 | 0.37 |
| C3 (mg/dL) | −2.53 (−5.07; 0.02) | −0.40 (−2.69; 1.89) |  | 0.22 | 0.22 |
| C4 (mg/dL) | 0.23 (−0.50; 0.97) | −0.20 (−0.77; 0.38) |  | 0.37 | 0.74 |
| Serum leptin (ng/mL) | −0.71 (−1.53; 0.11) | 0.77 (−0.14; 1.68) |  | 0.02 | 0.51 |
| Serum non-cholesterol sterols |  |  |  |  |  |
| Lathosterol (μg/mL) | −0.07 (−0.26; 0.12) | 0.01 (−0.14; 0.17) |  | 0.51 | 0.55 |
| Campesterol (μg/mL) | 0.21 (−0.01; 0.44) | 0.06 (−0.23; 0.35) |  | 0.40 | 0.12 |
| Sitosterol (μg/mL) | 0.19 (0.06; 0.32) | 0.15 (0.00; 0.29) |  | 0.64 | 0.14 |
| Cholestanol (μg/mL) | 0.02 (−0.09; 0.13) | 0.06 (−0.04; 0.16) |  | 0.56 | 0.59 |
| Serum fatty acids |  |  |  |  |  |
| Dihomo-γ-linolenic acid  (μg/mL) | −5.22 (−7.55; −2.88) | −2.67 (−4.72; −0.63) |  | 0.11 | 0.09 |
| AA (μg/mL) | 2.79 (−4.66; 10.2) | −2.43 (−8.18; 3.33) |  | 0.28 | 0.61 |
| EPA (μg/mL) | −7.88 (−13.8; −1.99) | −7.61 (−12.7; −2.51) |  | 0.95 | 0.73 |
| DHA (μg/mL) | −15.2 (−22.9; −7.61) | −12.4 (−18.1; −6.70) |  | 0.56 | 0.30 |
| EPA/AA ratio | −0.04 (−0.06; −0.01) | −0.03 (−0.06; −0.01) |  | 0.86 | 0.78 |
| Serum ketones |  |  |  |  |  |
| Acetoacetic acid (μmoL/L) | 7.72 (2.71; 12.7) | 0.28 (−3.40; 3.96) |  | 0.02 | 0.25 |
| 3-OH butyric acid  (μmoL/L) | 41.8 (18.7; 64.9) | 8.48 (−14.6; 31.6) |  | 0.05 | 0.83 |
| Total ketones (μmoL/L) | 49.5 (21.7; 77.3) | 8.78 (−17.6; 35.2) |  | 0.04 | 0.77 |

Follow-up measurements were done at 1, 3, and 6 months.

*, Based on a mixed-model repeated-measures analysis adjusting for sex, age, and baseline value of a parameter of interest.

†, *P* < 0.05 indicates the statistical significance for the between-treatment difference and treatment-month interaction.

AA, arachidonic acid; AST, aspartate aminotransferase; ALT alanine aminotransferase; ; CI, confidence interval; CPK, creatine phosphokinase; DHA, docosahexaenoic acid; EPA, eicosapentaenoic acid; GGT, gamma-glutamyl transferase; LDH, lactate dehydrogenase.

**Table S5. Adverse events reported during treatment**

| Adverse event | Ipragliflozin (n = 80) | Sitagliptin (n = 91) | *P** |
| --- | --- | --- | --- |
| Deterioration of diabetes | 1 | 2 | 1.00 |
| Diabetic nephropathy | 1 | 2 | 1.00 |
| Elevation in blood ketones | 4 | 0 | 0.046 |
| Pollakiuria | 2 | 0 | 0.22 |
| Thirst | 1 | 0 | 0.47 |
| Urinary infection | 1 | 0 | 0.47 |
| Skin disease† | 4 | 0 | 0.046 |
| Others‡ | 6 | 3 | 0.31 |
| Total | 20 | 7 | 0.003 |

*, Based on the Fisher’s exact test (two-sided).

†, 4 patients in the ipragliflozin group reported 4 events: eczema, contact dermatitis, skin eruption, and pruritis of the genital region.

‡, 6 patients in the ipragliflozin group reported 9 episodes: 2 episodes of herpes labialis and pollen allergy, 2 episodes of common cold and enhanced appetite, deterioration of angina pectoris, diarrhea, periarthritis of the shoulder, bruise of the ribs, cholelithiasis/cholecystitis; 3 patients in the sitagliptin group reported 4 episodes: hypertension and hyperuricemia, deterioration in dyslipidemia, and bone fracture of the hand finger.
